# Effective health care for older people resident in care homes: the optimal study protocol for realist review

**DOI:** 10.1186/2046-4053-3-49

**Published:** 2014-05-24

**Authors:** Claire Goodman, Adam L Gordon, Finbarr Martin, Sue L Davies, Steve Iliffe, Clive Bowman, Justine Schneider, Julienne Meyer, Christina Victor, Heather Gage, John RF Gladman, Tom Dening

**Affiliations:** 1 College Lane, University of Hertfordshire, Hatfield, Herts AL10 9AB, UK; 2 Kings Meadow Campus, University of Nottingham, Lenton Lane, Nottingham NG7 2NR, UK; 3 Kings College London, Strand, London WC2R 2LS, UK; 4 University College London, Gower St, London WC1E 6BT, UK; 5 City University, Northampton Square, London EC1V 0HB, UK; 6 Uxbridge Campus, Kingston Lane, Brunel University, Uxbridge, Middlesex UB8 3PH, UK; 7 University of Surrey, Guildford, Surrey GU2 7X, UK

**Keywords:** Residential facilities, Primary care, Older people, Health services, Realist review

## Abstract

**Background:**

Care homes in the UK rely on general practice for access to specialist medical and nursing care as well as referral to therapists and secondary care. Service delivery to care homes is highly variable in both quantity and quality. This variability is also evident in the commissioning and organisation of care home-specific services that range from the payment of incentives to general practitioners (GPs) to visit care homes, to the creation of care home specialist teams and outreach services run by geriatricians. No primary studies or systematic reviews have robustly evaluated the impact of these different approaches on organisation and resident-level outcomes. Our aim is to identify factors which may explain the perceived or demonstrated effectiveness of programmes to improve health-related outcomes in older people living in care homes.

**Methods/Design:**

A realist review approach will be used to develop a theoretical understanding of what works when, why and in what circumstances. Elements of service models of interest include those that focus on assessment and management of residents’ health, those that use strategies to encourage closer working between visiting health care providers and care home staff, and those that address system-wide issues about access to assessment and treatment. These will include studies on continence, dignity, and speech and language assessment as well as interventions to promote person centred dementia care, improve strength and mobility, and nutrition. The impact of these interventions and their different mechanisms will be considered in relation to five key outcomes: residents’ medication use, use of out of hours’ services, hospital admissions (including use of Accident and Emergency) and length of hospital stay, costs and user satisfaction. An iterative three-stage approach will be undertaken that is stakeholder-driven and optimises the knowledge and networks of the research team.

**Discussion:**

This realist review will explore why and for whom different approaches to providing health care to residents in care homes improves access to health care in the five areas of interest. It will inform commissioning decisions and be the basis for further research. This systematic review protocol is registered on the PROSPERO database reference number: CRD42014009112.

## Background

In the UK, long-term continuing care for older people is principally provided by independently owned (small, medium or large businesses and charities) care homes. The typical resident is female, aged 85 years or older, and in the last years of their life. The majority of care home residents have dementia and take seven or more medications. Many live with depression, mobility problems and pain [1-3]. Residents rely on primary health care services, for medical and nursing support, and access to specialist services.

There is a good understanding of the barriers and facilitators influencing how health care services work with care homes and evidence from a number of sources suggesting that care homes’ access to NHS services is erratic and inequitable [4-9]. Evidence about interventions that will redress these inequalities is less well established [3]. A review by Szczepura *et al*. [10] concluded that medical care for care home residents could be improved by making it more proactive. However, it remains unclear what approaches or key elements of practice are likely to sustain effective patterns of working between health services and care homes [1,11].

### Health care delivery to care homes as a complex intervention

There are numerous approaches to providing health care for this population. General practitioners have a statutory obligation towards care home residents registered with their practices but fulfil this obligation in various ways. Some care homes allow residents to choose their GP, whilst others contract with one practice. In addition to routine GP services, there are payment schemes for enhanced GP services, outreach clinics, care home specialist nurses or support teams, pharmacist-led services, designated NHS hospital beds and enhanced services [12,10-19]. This variety of services, coupled with the heterogeneity of care homes, suggest that it is unlikely that a single model of health service delivery can be similarly effective for all residents in all settings. The value of using a realist approach is that it gives equal consideration to the impact of the context on the outcomes of interest. A realist review is different to an empirically focused review as it is iterative and theory-driven. We suggest that it is, therefore, more useful to explain how and why different models of care delivery and their constituent parts are more or less effective in different circumstances or for different resident groups.

### Objectives and focus of review

The principal aim of this review is to understand the effectiveness of the various programmes focussed upon improving health care-related outcomes for people living in care homes. We propose to identify, map, and test the features or mechanisms of different approaches to health care provision to care homes in terms of six key outcomes that operate at the resident, service delivery or organisational level of care. These outcomes are: 1) residents’ medication use, 2) use of out of hours’ services, 3) hospital admissions (including use of Accident and Emergency), 4) length of hospital stay, 5) costs, and 6) user satisfaction. These are outcomes which are important to NHS commissioners and service providers but are also outcomes where care home residents differ substantially from older people living at home. Care home residents use out of hours services frequently and, when admitted to hospital, tend to have either very short or long stays [3]. Polypharmacy and prescribing and dispensing errors are common [20]. The relevance of the lived experience and service satisfaction of both care home residents, and professional and family carers, was identified by earlier work by the team [21].

The objectives at the outset of the research are to:

1. Identify which (elements of the) interventions could potentially be effective, how they work, on what range of outcomes, when, why and for whom.

2. Identify the barriers and facilitators to the acceptability, uptake, and implementation of arrangements designed to improve access to health care and health outcomes.

3. Establish what evidence there is on the feasibility of these arrangements and (if possible) their costs.

## Methods/design

The provision of health care to care homes is complicated, relying on multiple contributors over extended periods of time where uptake and use of resources can vary widely depending on individual needs. Realist synthesis is a systematic, theory-driven approach for making sense of diverse evidence about complex interventions applied in different settings [22-26]. To achieve this, a realist review brings together multiple sources of evidence to develop a programme theory of the intervention in question. Programme theories are possible explanations for the way in which particular interventions are thought to work and they describe the way in which change occurs because of an intervention. Realism understands causation, as working through mechanisms that operate, or not, according to context [27]. Realist approaches build plausible, evidenced explanations of observed outcomes, often for complex social interventions that have multiple components, operate across multiple sites, and involve multiple actors or agents [28,29]. The underlying premise is that the observed ‘demi-regular patterns’ of interactions between the components that make up complex interventions can be explained by mid-range theories. The iterative process of the review involves testing and re-testing those theories that are thought to work against the observations reported in each intervention included in the review [30].

**Figure 1:**
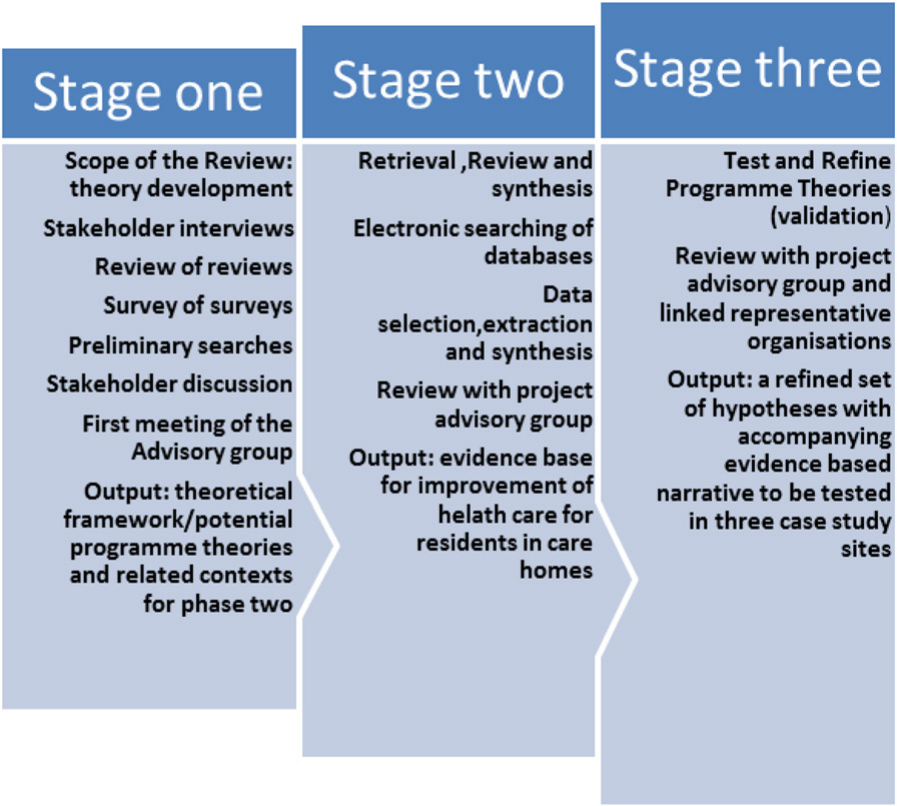
Realist review optimal health care for care home residents.

For the Optimal study, we propose the realist review will take a three-stage approach that is informed by key stakeholders (commissioners, care home and health care providers, residents and regulators) and that optimises the knowledge and networks of the research team (Figure 1).

### Stage one: defining the scope of the review: concept mining and theory development

To address objectives 1 and 2 and develop programme theories of how models of health care delivery for care homes work, we will focus on the contextual conditions and mechanisms that influence how services are provided to care homes in general, and specifically against the six outcomes. To guide the review process, stage one will develop an explanatory model and associated candidate programme theories of what works for care home residents, in what context and with what outcomes. This will be done through a survey of surveys, a review of reviews and stakeholder interviews.

### Survey of surveys

In the UK, care homes are regular users of NHS services. As many as thirty separate services may be linked to care homes, although, often only two or four services provide the core and ongoing support [18]. To capture findings that reflect the current organisation of health and are comparable with earlier survey work [5,11,31], we will focus on surveys of health care provision to care homes in the UK, completed since 2008. This will include surveys by professional organisations, care home providers, the regulator and health service researchers. We will also review professional literature on initiatives providing health care to care homes (for example, news about the development of new services and health-related initiatives for care homes). This will provide a contemporary account of how health care services are organised for care homes.

### Review of reviews

A review of published reviews on health service working with care homes will consider how mechanisms of service provision have been linked theoretically or empirically to the five outcomes of interest. We will search for relevant publications and reports using methods that draw on principles of systematic review methodology: document retrieval, review and scrutiny by multiple researchers, information retrieval and analysis against the research questions [32].

### Stakeholder interviews

To complete the scoping of the review and complement the national picture of provision, we will carry out stakeholder interviews about necessary preconditions for improving health care for older people resident in care homes. Stakeholders will be purposively selected on the basis of their understanding and experience of commissioning health care for care homes, or as providers and recipients of health care at the organisational, service and resident level of care. To capture a range of experience that reflects regional, historical and organisational differences, we will recruit representatives of resident and relatives (up to 5), NHS commissioners (n = 3), senior managers from care home organisations (n = 5), local authorities (n = 3) and regulators (n = 2). To ensure a breadth of older people’s views about health care and experience is captured, we will complement new resident interviews with a secondary data analysis of 34 resident interviews from an earlier study [21]. This study had investigated integrated working between primary health care and care homes, interviewing residents about their health and the health services they received. Analysing these interviews will provide insight regarding what residents thought was important about the health services they received and how they perceived effectiveness. To ensure transparency of approach and an audit trail, transcriptions of interviews and structured field notes on suggestions and decision-making processes about which sources of evidence were linked to which strands of theoretical development will be maintained [26]. The interview element of the study was reviewed and supported by the university ethics committee reference: NMSCC/12/12/2/A. All participants will be asked to provide written consent.

Stage one will provide a narrative account of how services are delivered in England, what is perceived by the different sources and informants to support effective working, methods of funding, frequency of provision and location. The project advisory group of care home providers, health service professionals, user representatives and service commissioners will review, debate and refine stage one findings for relevance and validity. Emergent theories of what is perceived as key to effective working in care homes will inform the interrogation of databases and relevant websites in stage two. They will provide an account of the behaviour and perceived interrelationships of the processes that are responsible for achieving change [33] and operational definitions to inform how they may be recognised and tracked within different models of service delivery to care homes. This will enable us to produce a list of propositions of the key mechanisms or features of service delivery and their different configurations, ranked according to the amount and quality of evidence available that are believed to have most influence on the outcomes of interest. These cannot be specified with precision in advance but they might be expected to include: patterns of working that support shared decision-making, systems that encourage evidence of regular review of residents; access to a range of services; and expectations of collaborative working (whether promoted through financial incentives, clinical interest, or regulatory expectations). This will provide a theoretical/conceptual framework and associated candidate programme theories that will inform the remainder of the review process.

### Stage two: theory refinement/testing

This will address objective two, and test the relevance and rigour of emerging findings from stage one by drawing comparison with the broader literature. A literature review will be undertaken. The findings, key themes, questions and approaches identified in stage one will be used to structure the data extraction forms and identify the key questions or hypotheses that the review will address. The review process will make explicit the processes and players involved in providing the services and the preferred mechanisms that cut across different models of service delivery and their influence on the five outcomes of interest. In keeping with published guidelines on realist review (RAMESES), equal consideration will be paid to negative and positive outcomes and recurring patterns in accounts of what works, when and with what outcomes.

### Searching process

We will search for relevant publications and reports using methods that draw on principles of systematic review methodology as described earlier. In addition we will search the grey literature using Internet search engines using search terms that offer maximum coverage, such as ‘care homes health care’ , ‘older people health care homes’ , ‘health service provision care homes’. We have used this method to successfully locate organisation-specific documents [1]. Relevant evidence will also exist in unpublished form, for example, care pathways, care home policy documents and service-based evaluations.

We will therefore seek to maximise opportunities to identify this literature through our consultations and interviews in phase one, through the experience of our research team and through our project steering committee. We will also request information through primary care and care home networks (for example, My Home Life Network, National Care Home Research and Development Forum, HCPOnet, PCRN, DeNDRoN, Age and Aging networks), and care home provider organisations and associations. We will search for published and unpublished literature. The project team will be involved in producing a list of relevant search terms to use in the following electronic databases:

### Pubmed

• CINAHL (Cumulative Index to Nursing & Allied Health Literature),

• The Cochrane Library including the Cochrane Database of Systematic Reviews, DARE (Database of Abstracts of Reviews of Effects), the HTA Database, NHS EED (NHS Economic Evaluation Database)

• Scopus

• SocAbs (Sociological Abstracts)

• ASSIA (Applied Social Sciences Abstract & Indexes)

• BiblioMap (The EPPI-Centre register of health promotion and public health research)

• Sirius, OpenGrey, Social Care Online, the National Research Register Archive, the National Institute of Health Research portfolio database Google and Google Scholar

In addition to the above electronic database searches, we will undertake the following lateral searches:

• Checking of reference lists from primary studies and relevant systematic reviews [34]

• Citation searches using the ‘Cited by’ option on WoS, Google Scholar and Scopus, and the ‘Related articles’ option on PubMed and WoS (‘Lateral Searching’) [35]

• Contact with experts to uncover grey literature (for example, DeNDRoN, National Library for Health Later Life Specialist Library, BGS, RCP, RCN)

• Contact with charities and user groups, residents and relatives associations

As we will have completed a review of reviews in stage one, our search will initially be limited to after 2006. The time limit reflects the fact that research in care homes is a relatively recent phenomenon [3] and progressive changes in the overall size, ownership and structure of the sector, coupled with the reorganisation of primary care, has altered the demographic characteristics of residents and how the two services work with each other [36-38]. In line with the iterative nature of realist synthesis methodology [39], the inclusion criteria will be refined in light of emerging data and the theoretical development in phase one. This refinement process will not be time limited.

We will include studies of any design including randomised controlled trials, controlled studies, effectiveness studies, uncontrolled studies, interrupted time series studies (ITS), cost effectiveness studies, process evaluations, surveys and qualitative studies of participants’ views and experiences of interventions. We will also include unpublished and grey literature, policy documents and information reported in specialist conferences or has been reported as successful, or innovative and promising, through stage one work that could provide a model for future practice or merit future evaluation.

Studies that focus on the following will be included:

• Residents in a care home with specific health needs/problems

• Studies of any intervention designed to improve the health status of care home residents that offer opportunities for transferable learning

• Studies that provide evidence on barriers and facilitators to the implementation and uptake of interventions in care homes generally (not confined to health care), that help with understanding of programme theories and logic, or that provide evidence on underlying theories that inform the particular approach and outcomes of interest

The review process will involve screening for relevance to the programme theories and data extraction forms will be developed to enable us to populate the evidence on context, mechanisms and the five outcomes of interest.

Search results from electronic databases will be imported to bibliographic reference management software and, where possible, duplicates deleted (Endnote). Documents from other sources will be manually recorded in the same Endnote Library.

### Selection and appraisal of documents and data extraction

The key test of a realist review is the relevance and rigour of the evidence [22]. The guiding principle for the review is that the quality of the evidence will be judged on its contribution to the building and testing of relevant theory. There may be evidence that has limited transferability to the NHS. For example, findings of research in the Netherlands are inevitably contingent on the unique context that all care homes have an on-site clinician medical director: in our review, findings will be interpreted accordingly.

Four reviewers will independently screen titles and abstracts to identify potentially relevant documents, which will be retrieved and assessed according to the inclusion criteria below. Disagreements will be resolved by discussion.

The programme theories being ‘tested’ through the review are made visible through the data extraction forms [24]. These forms will be developed using the programme theory, providing a template to interrogate the theories. If the evidence meets the test of relevance, data will be extracted using the form and then checked by a second member of the team. Quality assessment will be undertaken by two reviewers independently with any discrepancies resolved by discussion with other members of the project team. If appropriate, standardised quality assessment tools will be used [30] and whenever possible, the checking will be done by the team member who has the most relevant expertise (for example, interventions to reduce falls in care homes, impact of care home culture, uptake of innovation and so on).

### Stage 3: analysis and synthesis

This will address objective three and the analytical task is to synthesise the relationships between mechanisms (for example, underlying processes, structures, and entities), contexts (for example, conditions, types of setting, organisational configurations) and outcomes (that is intended and unintended consequences and impact). Rycroft-Malone *et al*. [24] have developed an approach to synthesis incorporating the work of Pawson [22] and principles of realist enquiry that includes individual reflection and team discussion to:

• Question the integrity of each theory

• Adjudicate between competing theories

• Consider the same theory in different settings

• Compare the stated theory with actual practice

Coded data from the studies will then be used to confirm, refute or refine the candidate theories. Where theories fail to explain the data, alternative theories will be sought. Table 1 summarises how this will be achieved, although in an iterative process results will be shared and discussed within the review team to ensure validity and consistency in the inferences and interpretations made. In particular, we will focus on prominent and recurrent patterns or demi-regularities of contexts and outcomes in the data and identify the mechanisms by which they have occurred [25].

**Table 1:** Analysis of evidence and development of propositions

	
1.	Using the hypotheses and questions generated from stage one, provided a detailed account of the processes and the underpinning mechanisms and contains factors identified. Organisation of extracted information into evidence tables.
2.	Data is themed to reflect the observed characteristics of health care working in and with care homes (taking account of confounding evidence).
3.	Connections between the extracted data and the themes is examined and repeatedly tested according to the evidence to build a cumulative picture of the possible context mechanism outcomes that have a basis in the available evidence.
4.	The development of propositions about different Context Mechanism Outcome (CMO) threads that the evidence indicates supports effective working in some or all of the five outcomes of interest.

The findings will be reviewed with the project team and project advisory group to develop statements of findings around which a narrative can be developed, summarising the links between context, mechanism and outcomes, and the evidence underpinning them. Equal consideration will be paid to negative and positive outcomes, the inclusion of different outcomes at different stages and inconsistencies in accounts of what works when and with what outcomes.

Attention will be given to what is revealed about resident, relative and care home staff priorities and if they coincide with NHS accounts and narratives of ‘good’ and ‘bad care’. This process of discussion will be carefully documented.

The research team, and the study steering committee, and where appropriate the networks they represent, will debate proposed mechanisms of interest supported (or refuted) by the evidence review The development of a consensus on how the study findings differentiate between the mechanisms of service delivery to care homes and the contexts that support or inhibit them will be key to this final analysis.

The conclusions of this review will inform the next phase of the Optimal study, which comprises case studies to test the identified mechanisms in a range of care homes to further clarify what works for whom in what circumstances.

## Discussion

The programme described will use realist review methods to understand a sector which is dynamic, changing and driven by complex financial, societal, political and organisational factors. A key challenge for the review will be to maintain a focus on what can be known about what supports access to health care for residents in care homes as opposed to strategies that may or may not improve residents’ wellbeing. This exhaustive review of accounts and evidence from residents of how health services work in and with care homes to achieve good outcomes will produce findings which, by describing the real-world setting, will be of relevance and use to NHS and social care commissioners and providers.

At the end of this work we will produce a briefing document for care homes and commissioners on what services are available, the accountability frameworks and models of care currently in place and how generalist and specialist services support care homes. This will include a critique of the range and quality of the available evidence recommendations for further work.

These findings will also provide the organising framework for phase 2 of the Optimal study which will seek to further establish the context, mechanism and outcomes frameworks described by considering their association with measured individual, care home and service- level outcomes.

The UK is not unique in having a complex long-term care sector which is in a state of continual flux. Realist evaluation methodology, as outlined here, offers a way of understanding the systems in and with which health services must operate-even when they are unable to directly influence the work that takes place in such settings.

## Abbreviations

BGS: British Geriatrics Society; CMO: Context Mechanism Outcomes DeNDRoN: Dementia and Neurodegenerative Diseases Research Network; NHS: National Health Service; PCRN: Primary Care Research Network; RAMESES: Realist And MEta-narrative Evidence Synthesis; RCN: Royal College of Nursing; RCP: Royal College of Physicians.

## Competing interests

The authors declare that they have no competing interests.

## Authors’ contributions

CG led the design and drafting of the review protocol. ALG JG, SI, FM, CB, TD, CV JS HG contributed to the design and drafting of the review protocol. CG scoped and designed the search strategy in collaboration with an information specialist JG, JM, CB, SLD, TD provided substantive topic-specific input that informed the revision and refinement of the review protocol. All authors read and approved the final manuscript.
